# Properties and Applications of Self-Healing Polymeric Materials: A Review

**DOI:** 10.3390/polym15224408

**Published:** 2023-11-14

**Authors:** Kiwon Choi, Ahyeon Noh, Jinsil Kim, Pyong Hwa Hong, Min Jae Ko, Sung Woo Hong

**Affiliations:** 1 Department of Chemical Engineering, Hanyang University, 222 Wangsimni-ro, Seongdong-gu, Seoul 04763, Republic of Korea; 2 Department of Chemical Engineering, University of Montreal, 2900 Edouard Montpeit Blvc, Montreal, QC H3T 1J4, Canada; 3 Green and Sustainable Materials R&D Department, Korea Institute of Industrial Technology (KITECH), 89 Yangdaegiro-gil, Ipjang-myeon, Seobuk-gu, Cheonan-si 31056, Chungcheongnam-do, Republic of Korea; 4 Department of Materials Science and Engineering, Korea University, 145 Anam-ro, Seongbuk-gu, Seoul 02841, Republic of Korea; 5 Convergence Research Center for Solutions to Electromagnetic Interference in Future-Mobility, Korea Institute of Science and Technology (KIST), 5, Hwarang-ro 14-gil, Seongbuk-gu, Seoul 02792, Republic of Korea

**Keywords:** self-healing, polymer, plastic, extrinsic, intrinsic

## Abstract

Self-healing polymeric materials, engineered to autonomously self-restore damages from external stimuli, are at the forefront of sustainable materials research. Their ability to maintain product quality and functionality and prolong product life plays a crucial role in mitigating the environmental burden of plastic waste. Historically, initial research on the development of self-healing materials has focused on extrinsic self-healing systems characterized by the integration of embedded healing agents. These studies have primarily focused on optimizing the release of healing agents and ensuring rapid self-healing capabilities. In contrast, recent advancements have shifted the focus towards intrinsic self-healing systems that utilize their inherent reactivity and interactions within the matrix. These systems offer the advantage of repeated self-healing over the same damaged zone, which is attributed to reversible chemical reactions and supramolecular interactions. This review offers a comprehensive perspective on extrinsic and intrinsic self-healing approaches and elucidates their unique properties and characteristics. Furthermore, various self-healing mechanisms are surveyed, and insights from cutting-edge studies are integrated.

## 1. Introduction

In recent years, substantial progress has been made in the development of polymeric materials, leading to accelerated growth in the associated industries and a rapid increase in plastic production and consumption [1,2]. This has resulted in significant plastic waste generation and caused environmental concerns [3,4,5,6,7,8]. One of the primary reasons for the disposal of polymers is their inability to maintain their initial appearance and functionality during use [9,10,11]. In such instances, the implementation of a self-healing system capable of autonomously repairing the damages caused by external stimuli, thereby extending the material lifespan, has proven to be an efficient approach for reducing polymeric waste [12,13,14].

The concept of self-healing is inspired by the regenerative abilities of living organisms that can naturally recover from damage [15,16,17,18]. Over the past decade, extensive research has been conducted on the development of self-healing properties in materials, resulting in significant advancements in this field [15,19,20,21,22,23,24]. Similar to living organisms, self-healing materials restore their characteristics and properties after damage. Self-healing systems can protect external layers. For example, they shield the delicate core of metallic materials from potential performance degradation resulting from physical damage [25,26]. In addition, they highlight their characteristics that contribute both to their self-healing properties and performance as a device [27,28]. As a result, they find widespread application as protective coating materials for electronic devices and vehicles, effectively extending their operational lifespan [29]. This is achieved by mitigating damage to their external appearance, which preserves the integrity of internal circuits and prevents cracks or scratches. Recent advancements have shown self-healing materials capable of achieving commendable electrochemical or thermal performances. However, most self-healing materials involve a trade-off between their properties and characteristics, which is a significant challenge to their widespread use in numerous practical applications. Therefore, the development of novel molecular designs for self-healing materials is imperative, not only to reduce plastic waste but also to extend the lifespan of materials [11,30,31].

Self-healing materials can be broadly categorized into intrinsic and extrinsic types, depending on the underlying principles of self-healing. Extrinsic self-healing materials rely on self-healing agents embedded within a matrix, which are dispersed in the form of capsules and vascular structures [32,33,34,35,36]. When materials sustain damage, the capsules or vascular structures rupture, releasing self-healing agents. These agents seal and repair the damaged regions. Extrinsic self-healing materials can facilitate rapid self-healing over a large area but are limited by the number of multiple self-healing cycles.

On the other hand, intrinsic self-healing materials utilize their unique reversible reactions and bonding capabilities, allowing them to specifically interact with each other within the matrix [15,16,24]. When damage occurs from external stimuli such as heat [11,12,14], water [37,38,39], and light [40,41,42], a reversible mechanism is triggered, facilitating the repairing process analogous to the operation of a zipper. Various reversible chemical reactions and supramolecular interactions, including disulfide metathesis [13,43,44,45,46,47,48], the Diels–Alder reaction [11,14,49,50,51], hydrogen bonding [12,52,53,54,55,56,57,58], and ionic interactions [37,59,60,61], serve as representative mechanisms in intrinsic self-healing materials. Theoretically, intrinsic self-healing materials offer the crucial advantage of performing unlimited self-healing cycles. However, intrinsic self-healing materials pose a significant challenge: the trade-off between mechanical strength and self-healing performance [12,54,61,62,63,64]. This trade-off arises from fundamental differences in the underlying concept of polymeric motion. Mechanical strength can be increased by enhancing the interactions between the polymeric chains [65]. However, the intensified polymeric interactions significantly reduce polymeric chain mobility, resulting in diminished self-healing capabilities.

This review comprehensively investigates extrinsic and intrinsic self-healing materials, focusing on their mechanisms and characteristics. Additionally, it addresses recent studies investigating this trade-off in conventional self-healing materials. Furthermore, this review offers an extensive comparative analysis of diverse extrinsic and intrinsic self-healing materials, contributing to a deeper understanding of their strengths and limitations in contemporary materials science.

## 2. Extrinsic Self-Healing

The extrinsic self-healing mechanism involves incorporating heterogeneous substances into the polymer matrix to fill areas where cracks have formed, irrespective of specific interactions in the matrix. This section comprehensively examines the advantages and disadvantages of extrinsic self-healing systems compared with intrinsic self-healing systems. A notable advantage of extrinsic self-healing materials is their rapid response to crack repair. When exposed to a catalyst or air, a self-healing agent encapsulated within the matrix undergoes a rapid chemical reaction, facilitating crack sealing. However, a limitation arises when cracks reoccur in the same area, as the consumed self-healing agent cannot be replenished, rendering multiple self-healing performances unfeasible. Consequently, extrinsic self-healing materials are most suited for emergencies such as severe damage in aircraft, automobiles, buildings, or wind turbine blades. On the other hand, due to their inherent heterogeneity, a distinct disadvantage is their inability to meet the requirements of applications demanding optical properties.

**Figure 1:**
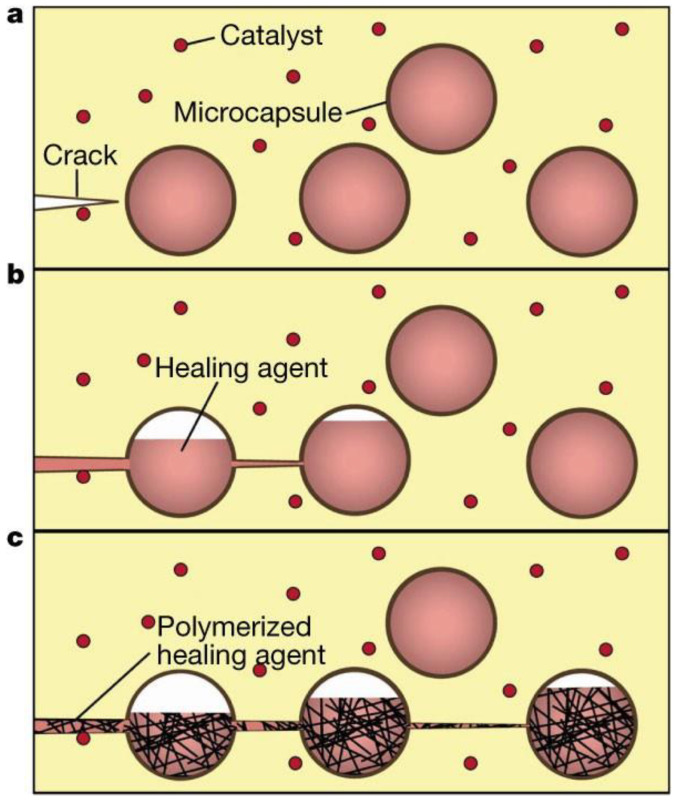
Schematic illustration of microencapsulated healing agents within a composite matrix integrated with catalysts for polymerizing the healing agents. (**a**) The matrix exhibits damage and cracks. (**b**) When cracks reach the microcapsules, capillary action releases the healing agents into the cracks. (**c**) Upon contact with the catalyst, the healing agents undergo polymerization, sealing the crack. Reproduced with permission [32], Copyright 2001, Springer Nature.

Extrinsic self-healing materials are exemplified by composites with capsule- and vascular-based internal carriers for self-healing, which typically contain polymerizable or cross-linkable monomers [15,32,33,34,35,36,66,67,68,69,70,71,72,73]. When composites are damaged, the protective shell of the capsular or vascular agent cracks, allowing monomers to be drawn into the crack through a capillary effect. The released monomers initiate polymerization or cross-linking upon contact with the catalysts dispersed throughout the matrix, thereby restoring the damaged region. The released monomers react with the catalysts dispersed in the matrix, inducing polymerization to recover the cracks. Figure 1 illustrates the mechanism of capsular-type self-healing [32]. Vascular-type self-healing operates as a capsular-type self-healing but employs a vascular form of encapsulation [69,72]. A representative example involves a rapid cross-linking polymerization facilitated by a catalyst. When a fracture occurs in a polymer matrix comprised of capsules containing dicyclopentadiene (DCPD) and Grubbs’ catalysts uniformly dispersed in proximity to those capsules, the rupture of the DCPD-filled capsules prompts a catalytic reaction with the dispersed catalysts, thereby leading to the effective polymerization process.

### 2.1. Capsular Type

White et al. reported a self-healing composite that utilized a dicyclopentadiene (DCPD) monomer as a healing agent in conjunction with a ruthenium-based Grubbs’ catalyst [32]. When the microcapsules within the composite ruptured, the DCPD was released into the cracked area. The Grubbs’ catalyst dispersed throughout the matrix facilitated the ring-opening reaction of DCPD, forming a highly cross-linked polymeric network. After self-healing, the self-healing efficiency was measured using a fracture test, which revealed a recovery of approximately 75% of the initial fracture load. However, the capsules had a diameter ranging from 50 to 200 μm, rendering them unsuitable for thin film applications.

To address these challenges, the research group refined their approach by introducing a sonication process, which resulted in smaller capsule sizes and increased capsule dispersion [33]. They not only embedded capsules within epoxy resin but also developed a capsular-type self-healing elastomer based on poly(dimethylsiloxane) (PDMS) [35]. Within the PDMS matrix consisting of platinum catalysts, two different capsules were incorporated: one containing vinyl-terminated PDMS and the other containing a PDMS copolymer with silane groups that the platinum catalysts could activate. When cracks occurred, the platinum-catalyzed hydrosilylation facilitated the formation of a cross-linked PDMS network. Nevertheless, it has been known that self-healing systems based on capsules are characterized by high production costs, inconsistent size distribution, and challenges in achieving efficient connectivity between capsules. This necessitates the development of alternative self-healing agents.

### 2.2. Vascular Type

**Figure 2:**
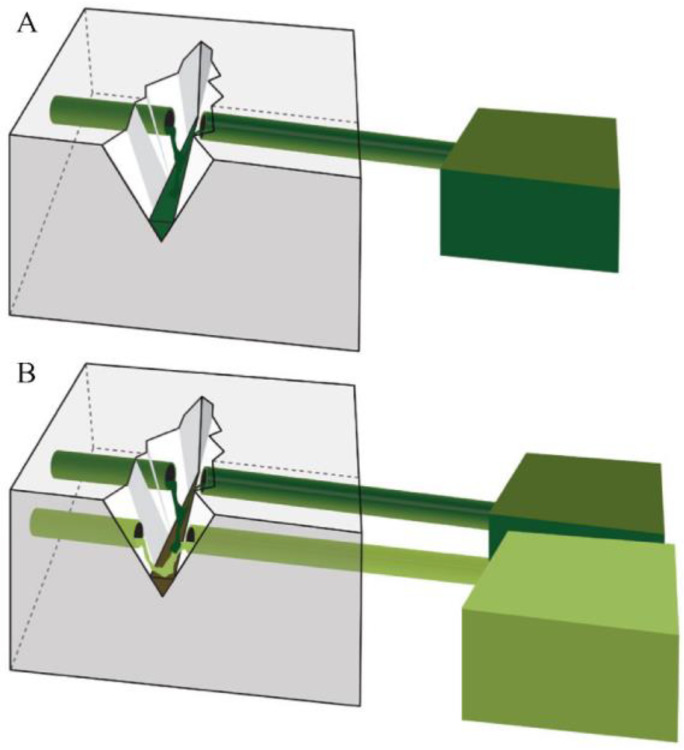
Illustration of vascular-type self-healing materials. Upon damage, healing agents are dispensed from the reservoir through gravitational, capillary, and hydrostatic forces. The vascular self-healing systems are depicted with single-channel (**A**) and multi-channel (**B**) designs. Reproduced with permission [73], Copyright 2013, MDPI.

Researchers have explored vascular-type self-healing systems to address the connectivity issue of capsular-type self-healing. The fundamental self-healing mechanism of vascular- and capsule-type materials is similar, as shown in Figure 2 [73]. However, vascular materials establish an interconnected three-dimensional network through the entanglement of vascular agents, enhancing the efficient diffusion of self-healing agents [66,67,68,69,70,71].

In a noteworthy example of vascular-type self-healing materials, An et al. investigated self-healing core-shell nanofibers based on PDMS [69]. These fibers featured polyacrylonitrile (PAN) as the outer shell, and vinyl-terminated PDMS and dimethyl-methyl hydrogen-siloxane were embedded via coaxial electrospinning. The resin and curing agent were released from the fibers and cross-linked after scratching the film. This study involved the evaluation of the self-healing properties through a corrosion test. An acetic acid solution was applied to the scratched film and deposited onto steel substrates. The film containing the self-healing fibers exhibited corrosion resistance owing to the self-healing process. Despite overcoming some of the limitations associated with capsular-type materials, vascular-type materials are still subject to the inherent constraints of extrinsic self-healing materials, which limits them to a single self-healing cycle.

## 3. Intrinsic Self-Healing

**Figure 3:**
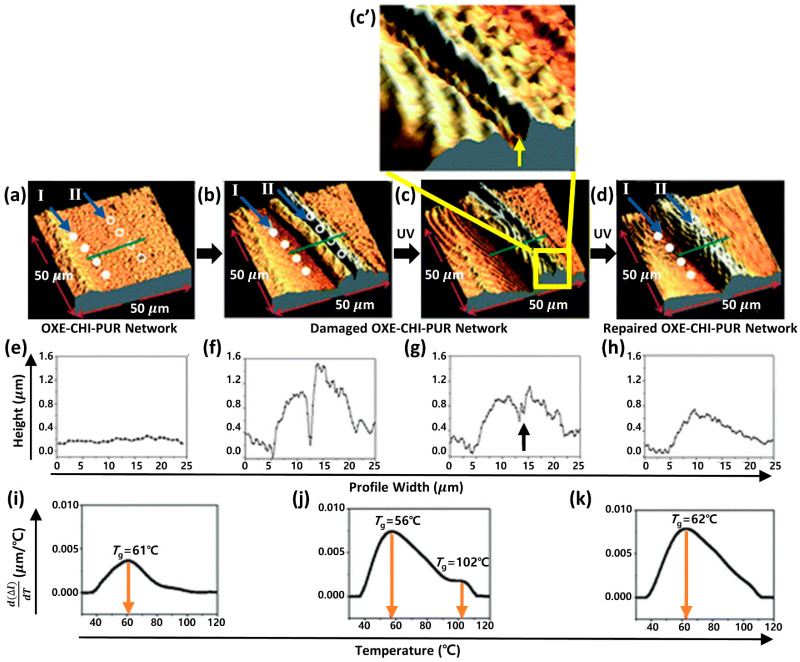
Atomic force microscopy (AFM) images of the OXE-CHI-PUR networks under various conditions: undamaged (**a**), damaged (**b**), UV-exposed (**c**,**c’**), and repaired (**d**), with the formulation ratio of HDI: PEG: OXE-CHI: DBTDL being 1.0: 1.33: 1.17 × 10^−4^: 2 × 10^−5^. The green line in (**a**) represents the direction of depth profiling, and I and II in (**a**) indicate the points for measuring thermal expansion before and after self-healing, respectively. Accompanying these are plots comparing height against width for undamaged (**e**), damaged (**f**), UV-exposed (**g**), and repaired (**h**) networks, alongside the d(Δl)/dT against temperature profiles (**i**–**k**) for the respective networks. Reproduced with permission [40], Copyright 2011, The Royal Society of Chemistry.

Intrinsic self-healing systems have emerged as a promising approach that enables multiple cycles of self-repair through unique reversible reactions or intermolecular interactions within the material. When a crack forms, these reversible interactions facilitate the gradual filling of the crack, starting from the deepest layers and progressing toward the surface, similar to the operation of a zipper (see Figure 3) [41,74,75]. Depending on whether the underlying mechanism involves covalent or non-covalent bonding, intrinsic self-healing can be categorized as chemical reaction- or supramolecular interaction-based self-healing [15,24]. Notable examples of chemical reaction-based mechanisms include metathesis [45,46,47,48,49,50,51] and exchange reactions [76,77,78,79,80,81]. Most supramolecular interactions can be employed for the typical non-covalent bonding to achieve self-healing [82]. High mobility and low glass transition temperatures are favorable for achieving high self-healing efficiency. These properties facilitate molecular motion, leading to improved wetting and enhanced interaction opportunities, thereby enhancing the self-healing capability of the material.

It is noted that extrinsic systems depend on embedded capsules or vascular networks. When the material sustains damage, these embedded containers release healing agents that facilitate repair through processes involving polymerization or chemical reactions. However, achieving repeated healing at the same location presents inherent challenges due to the limited quantity of healing agents within the container. Furthermore, the healing capacity diminishes immediately after the depletion of the healing agent.

Conversely, intrinsic systems utilize reversible or supramolecular interactions within the matrix, enabling them to repeatedly self-heal the same damaged areas. It is also noted that, unlike intrinsic systems, extrinsic systems encounter difficulty in preserving their initial optical properties after the self-healing process. This arises from significant color and refractive-index differences between the intact matrix and the healed region. For these reasons, intrinsic systems are considered a more suitable solution for display applications requiring excellent optical properties.

### 3.1. Self-Healing Based on Chemical Reactions

The fundamental approach to self-healing based on chemical reactions involves repetitive disconnection and reformation of reversible bonds. When damage occurs, these reversible bonds, which are inherently weaker than the irreversible covalent bonds, are broken intentionally instead of undergoing irreversible bond fractures. Subsequently, the broken bonds can be reconstituted under specific self-healing conditions, resulting in the recovery of the damaged area. Several reversible reactions have been explored to implement self-healing capabilities, including the Diels–Alder reaction, disulfide reaction, [2 + 2] cycloaddition reaction, siloxane chain exchange, trithiocarbonate reshuffling, and boronic ester exchange reaction.

Briefly, the incorporation of reversible reactions, including Diels–Alder, disulfide exchange, and boronic ester exchange reactions, has been widely studied for advancing functional self-healing materials. These dynamic reversible chemical processes enable materials to repair damage autonomously. The reversibility of these reactions supports repetitive healing cycles, thereby prolonging the functional lifespan of the materials and addressing the constraints faced by extrinsic self-healing systems. However, the mechanical strength is compromised when covalent bonds within the material are transformed into reversible bonds. Current research efforts have been focused on overcoming the limitations of self-healing materials based on chemical reactions.

#### 3.1.1. Diels–Alder Reactions

The mechanism involving thermally cross-linked polymers through the Diels–Alder reaction (DA) can serve as the driving force for self-healing [49]. The DA reaction is a chemical cross-linking method involving a reversible interaction between a diene and a dienophile, forming a cyclohexene bond within a specific temperature range. The cyclohexene bond can be dissociated and reformed reversibly, depending on temperature. In the context of self-healing, when heat is applied to a cut surface the cyclohexene structure is disrupted at 110 °C, and the two cut sections are drawn together as the diene and dienophile groups undergo a reaction at 70 °C While the DA reaction has been extensively explored for self-healing applications [11,14,49,50,51], it is constrained by its requirement for relatively high temperatures due to its activation energy.

**Figure 4:**
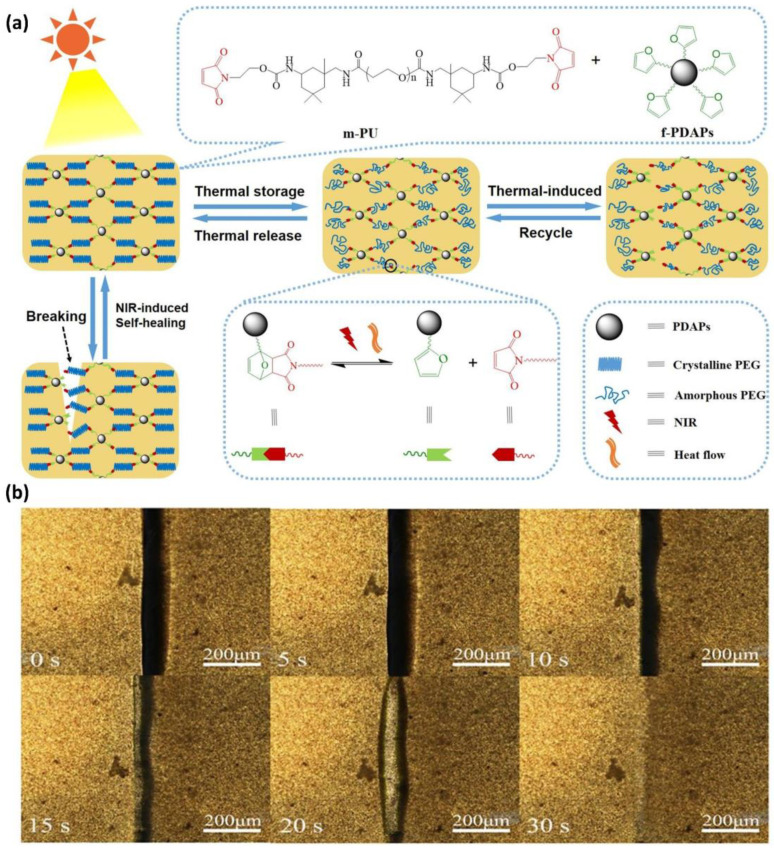
(**a**) Schematic representation of the self-healing mechanism in composites utilizing the DA reaction under NIR irradiation. (**b**) Optical microscopy images show the self-healing process of a DA-infused polymer exposed to NIR irradiation (output power: 0.5 W/cm^2^) over 30 s. Reproduced with permission [50], Copyright 2020, Elsevier B.V.

Several studies have been conducted on achieving self-healing under mild conditions. According to Yang et al., a self-healing polymer based on polyurethane incorporating the DA reaction achieved an impressive self-healing efficiency of 93.1% without requiring elevated temperatures [50]. Linear polyurethane was synthesized with maleimide groups at both ends, and furan-modified polydopamine particles were employed. Remarkably, this system achieved high self-healing efficiency by indirectly generating heat in the polydopamine via near-infrared (NIR) irradiation, eliminating the necessity for direct heating (Figure 4). Despite this innovative self-healing method, it is essential to note that the DA self-healing system still relies on thermal energy exceeding the activation energy of the DA reaction.

In the pursuit of multifunctionality, Lian et al. introduced 9,10-dihydro-9-oxa-10-phosphaphenanthrene-10-oxide as a flame retardant incorporated into a diene, thereby achieving a material with dual capabilities of self-healing and flame retardancy [83]. Although the resulting elastomer required elevated thermal conditions (150 °C) to facilitate self-healing, the introduced phosphorous-based structure exhibited remarkable thermal responsiveness, undergoing a transformative process that converted it into polyphosphoric acid-like substances. This transformation promoted the dehydration of the epoxy material into carbon and established a robust carbon layer, ensuring effective flame-retardant properties. This approach represents a significant advancement in developing multifunctional self-healing materials, thus broadening their range of potential applications.

#### 3.1.2. Disulfide Reactions

To address this challenge, researchers have turned to disulfide groups [13,43,44,45,46,47,48]. The disulfide groups are renowned for their metathesis exchange reactions [47]. These bonds are capable of reversible disconnection and regeneration as one sulfur atom is exchanged with a neighboring sulfur atom within a disulfide bond. Furthermore, polymers incorporating disulfide groups typically exhibit low glass transition temperatures, promoting molecular mobility at moderate temperatures and enhancing their self-healing capabilities. The incorporation of disulfide groups has emerged as a promising strategy for achieving self-healing materials.

**Figure 5:**
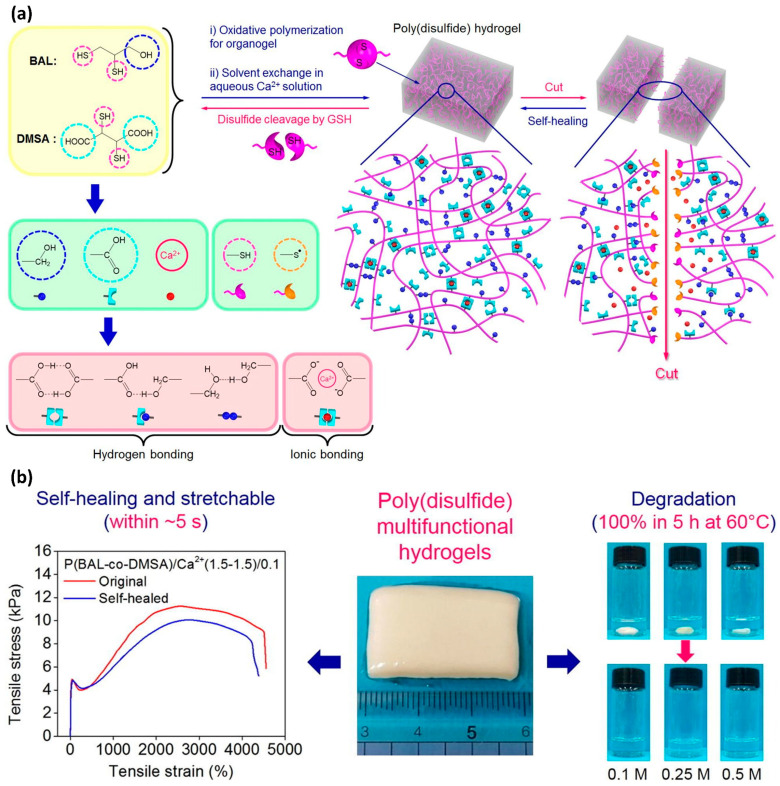
(**a**) Chemical structure and graphical representation of the self-healing mechanism in a hydrogel containing disulfide bonds. The thiol groups on the cleaved surfaces facilitate the self-healing process. (**b**) Tensile stress–strain graphs comparing the elasticity of both the original and self-healed hydrogel samples. Reproduced with permission [48], Copyright 2020, Elsevier B.V.

Tran et al. developed a self-healing hydrogel with an exceptional 5000% elongation at break compared with its pristine state, achieving a remarkable 4445% stretchability after self-healing through the introduction of disulfide groups (Figure 5) [48]. The self-healing process was completed within 10 s at ambient temperature. Despite these promising results, disulfide-based self-healing systems still face several challenges. One notable issue is the distinctive yellow color of sulfur-containing materials. A more significant concern is the decline in mechanical strength, which is a common drawback observed in many self-healing materials.

Regarding properties such as elongation at break and self-healing capability, reversible but relatively weak bonds, such as disulfide bonds, are advantageous because of their sacrificial bond rupture and reformation. Conversely, the mechanical strength, including tensile strength and Young’s modulus, decreased as the proportion of the rigid covalent network decreased. This trade-off between self-healing and mechanical properties has long been challenging for self-healing materials.

**Figure 6:**
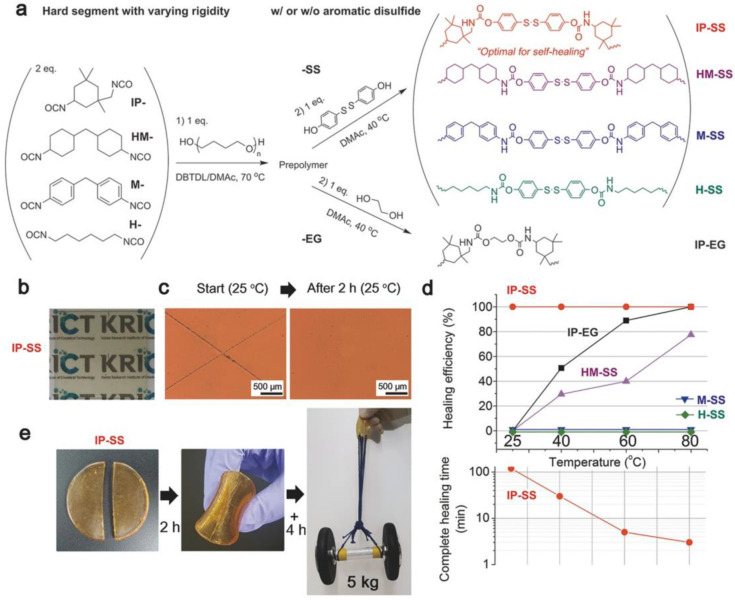
(**a**) Chemical structures of thermoplastic polyurethane (TPU) featuring different core structures with and without disulfide groups. (**b**) A photograph illustrating the clarity of the TPU film. (**c**) Optical microscopy images of an X-shaped scratch on the TPU film before and after 2 h of self-healing at 25 °C. (**d**, **Top**) A comparative study of self-healing efficiencies in TPU films after 2 h at 25 °C and 1 h at 40, 60, and 80 °C. (**d**, **Bottom**) Time required for complete self-healing of the scratch. (**e**) A TPU film, once cut and then rejoined, undergoes self-healing for 2 h (+4 h) at 25 °C and is subsequently tested by lifting a 5 kg dumbbell. Reproduced with permission [45], Copyright 2017, WILEY-VCH.

However, recent advancements have shown promise in overcoming this trade-off. For instance, a disulfide-containing elastomer demonstrated rapid self-healing capabilities, achieving 75% healing within 2 h, and remarkable toughness, reaching up to 26.9 MJ/m^3^ (Figure 6) [45]. Kim et al. incorporated disulfide groups as monomers into thermoplastic polyurethane (TPU) to achieve the highest tensile strength. Even after a cut and self-healing process, this material withstood a 5 kg dumbbell lifting test. The self-healing process was conducted under mild conditions, specifically at 25 °C (room temperature) over 6 h. To mitigate the trade-off mentioned above in the relationship, this group harnessed the ability of TPU to enhance mechanical strength by fine-tuning the chemical structure and the ratio of the hard segment.

#### 3.1.3. Boronic Ester Exchange Reactions

In contrast to heat-induced self-healing mechanisms, the boronic ester exchange reaction is a water-triggered reversible reaction [74,84,85,86,87]. This reaction occurs between boronic acid and 1,2-diols or 1,3-diols and results in the formation of boronic ester bonds; the equilibrium process is influenced by pH and moisture. Under humid conditions, these bonds undergo repeated esterification, hydrolysis, and re-esterification. During these cycles, exchange reactions occur between neighboring boronic ester bonds, pulling the severed sections together to achieve self-healing.

Using this mechanism, Cash et al. introduced a water-triggered self-healing boronic ester network [84]. This network consists of boronic ester bonds that connect the polymer chains and many free-dangling diols linked to the polymer backbone. Self-healing was accomplished by subjecting the material to 85% humidity for 3 days, facilitating boronic ester exchange and re-esterification between the hydrolyzed boronic ester groups and adjacent diols. Although the self-healing mechanism involving water is intriguing, the unusually high humidity level of 85% and the time-intensive nature of the process limit it.

**Figure 7:**
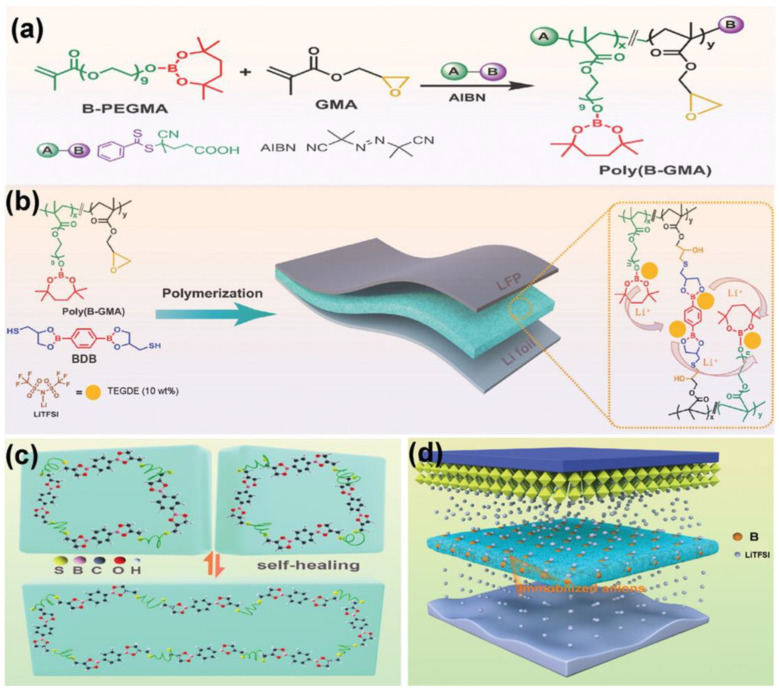
(**a**) The poly(B-GMA) synthesis process using RAFT polymerization. The polymerization occurs between poly(ethylene glycol) methacrylate with boronic ester (B-PEGMA) and glycidyl methacrylate (GMA) to produce poly(B-GMA). (**b**) Graphical illustration of the creation of the dynamic boronic ester-based self-healing polymer electrolyte (DB-SHPE). The thiol group in dimercapto-borate crosslinker (BDB) triggers the ring-opening reaction of the epoxy group in poly(B-GMA). (**c**) Schematic representation of the self-healing mechanism driven by boronic ester exchange reactions within the DB-SHPE. (**d**) Role of the boron groups in DB-SHPE in facilitating uniform deposition of Li ions in lithium metal batteries. Reproduced with permission [78], Copyright 2023, Wiley-VCH GmbH.

Figure 7 shows that adding boronic ester groups imparts self-healing capabilities and enhances lithium ionic conductivity when employed as a polymer electrolyte [78]. Furthermore, the unique trans-esterification of the boronic ester groups enables recyclability. These chemical bond-based self-healing materials exhibit self-healing performance when exposed to various conditions, including heat, light, or humidity. However, it is essential to note that most self-healing materials relying on chemical bond mechanisms necessitate relatively high temperatures above their activation energy, limiting their applicability. Additionally, the network of chemical reaction-based self-healing systems relies on relatively weaker reversible bonds than covalent bonds, which can reduce the mechanical strength.

### 3.2. Self-Healing Based on Supramolecular Interactions

Supramolecular reversible interactions offer promising solutions to address the challenges associated with chemical reaction-based self-healing. This approach involves the construction of a self-healing network that incorporates a functional self-healing moiety while preserving the existing polymer backbone. The polymer backbone contributes mechanical strength, whereas the flexible self-healing moiety facilitates self-healing. Elastomers such as polyurethane or PDMS are commonly employed as polymer backbones because their high elasticity and mobility maximize the self-healing effect [79].

Dynamic supramolecular interactions, including host–guest interactions, metal–ligand interactions [88,89,90,91], ionic interactions [37,59,60,61], and hydrogen bonding [12,52,53,54,55,56,57,58], have been utilized as self-healing moieties. These interactions are advantageous because they can occur under mild conditions, thereby enhancing the applicability of self-healing materials.

#### 3.2.1. Host–Guest Interactions

Host–guest interactions exhibit robust binding affinities and maintain fixed geometries and orientations, often in response to environmental factors such as pH or other stimuli [80,81,82]. Cyclodextrin (CD) has emerged as a widely employed host molecule owing to its relatively hydrophobic internal cavity, which readily accommodates the hydrophobic binding sites of guest molecules. Nakahata et al. reported the development of supramolecular hydrogels, redox reactions, and self-healing capabilities using CD [80]. CDs are environmentally friendly and can be used in diverse fields. A supramolecular hydrogel was developed by blending poly(acrylic acid) (pAA) with β-CD as the host polymer and pAA with ferrocene as the guest polymer.

**Figure 8:**
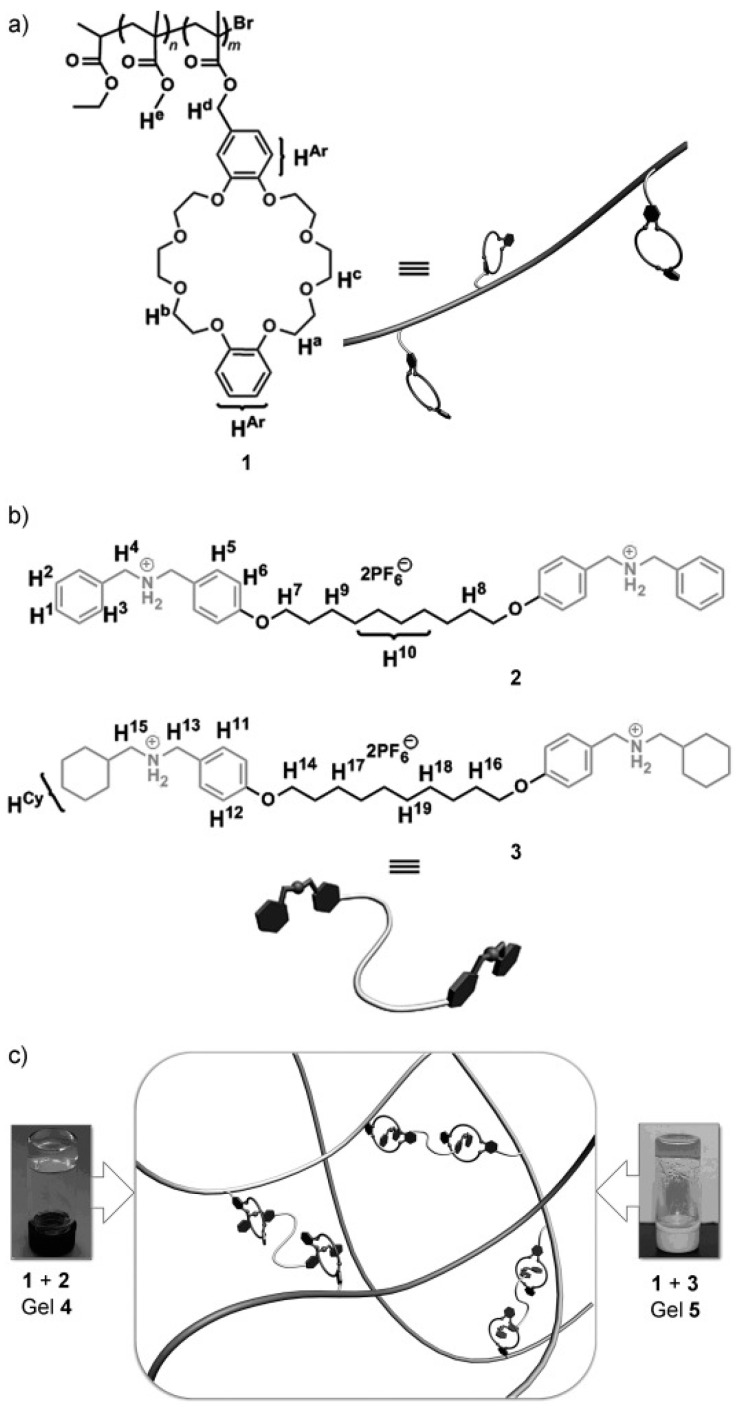
Chemical structures of (**a**) the host polymer 1 and (**b**) guest molecules, specifically cross-linkers 2 and 3. (**c**) Supramolecular gels 4 and 5 are produced by combining the host polymer with the guest molecules. Reproduced with permission [81], Copyright 2012, WILEY-VCH.

Additionally, redox stimuli were harnessed to modulate the self-healing properties by promoting re-adhesion between the cut surfaces [81]. Zhang et al. reported polymer gels formed by mixing poly(methyl methacrylate) (PMMA) with bisammonium cross-linking agents featuring different end structures, along with pendent dibenzo [24] crown-8 (DB24C8) units involved in host–guest interactions (Figure 8). These gels responded to pH stimuli and functioned as degradable materials with self-healing properties.

Recent advancements in self-healing materials involving host–guest interactions have found applications in drug delivery, leveraging their pH-responsive properties. Jiang et al. introduced an approach by incorporating adamantane and β-CD into cellulose, forming a self-healing hydrogel through host–guest interactions [92]. A noteworthy characteristic of this system is its ability to reattach the cut cross-section within an hour, a capability absent in cellulose lacking host–guest functionality. In addition to its rapid self-healing, the pH-responsive hydrogel exhibited controlled drug delivery properties, with dissociation occurring at low pH and coagulating at high pH. Importantly, this system demonstrated low toxicity, significantly advancing in developing self-healing materials for drug delivery applications. The unique combination of rapid self-healing and pH-responsive drug release holds promise for various biomedical and therapeutic applications.

#### 3.2.2. Metal–Ligand Interactions

**Figure 9:**
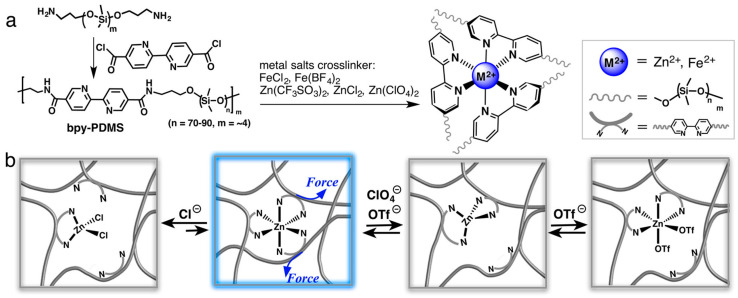
(**a**) Synthesis process for the polydimethylsiloxane (PDMS) cross-linked by metal cation. (**b**) Graphical representation of the hypothesized dynamic interactions among metal cations Zn^2+^, the ligand, and counteranions within the polymer systems when subjected to mechanical stress. Reproduced with permission [89], Copyright 2016, American Chemical Society.

Various dynamic metal–ligand interactions have been explored in the scientific literature to advance the development of self-healing materials [88,89,90,91]. As an illustration, Rao et al. presented cross-linked elastomers utilizing metal salts through metal–ligand coordination involving transition metal ions (Fe^2^⁺, Zn^2^⁺) and bipyridine moieties incorporated within the PDMS backbone (Figure 9) [89]. The selection of metal cations and pyridine moieties influences the coordination geometry and binding strength, consequently affecting the mechanical and electrical properties of the elastomer. The kinetic instability of metal–ligand coordination bonds determines their self-healing properties. For instance, the iron–ligand bond can be readily disrupted and reformed, facilitating the reversible unfolding and refolding of the polymer chains. Li et al. reported that the iron–ligand bond contributed to the remarkable elasticity and self-healing capability [88].

Numerous research groups have studied the interactions between transition metal ions (Fe(II), Ru(II), Ni(II), or Co(IV)) and polymer chains to achieve self-healing properties. The fundamental mechanism involves the interaction between the metal ion and ligand, usually pyridine-derivative groups, which draw the cut surfaces apart and restore them.

#### 3.2.3. Ionic Interactions

**Figure 10:**
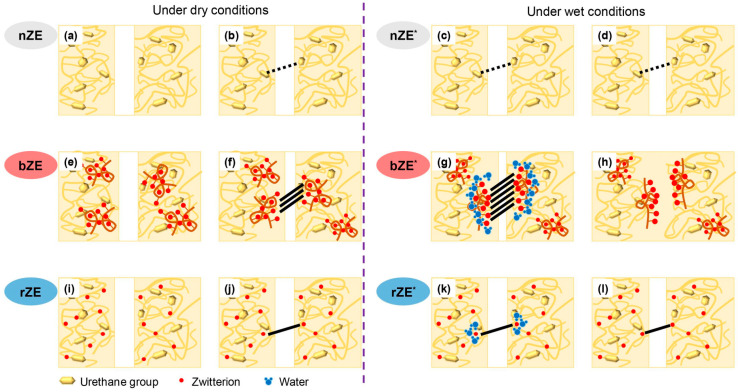
Schematic illustrations of the self-healing mechanisms of (**a**,**b**) nZE, (**e**,**f**) bZE, and (**i**,**j**) rZE under dry conditions; and (**c**,**d**) nZE*, (**g**,**h**) bZE*, and (**k**,**l**) rZE* under wet conditions (nZE: conventional elastomer with no zwitterions; bZE: Blend of conventional elastomer and zwitterionic elastomer; rZE: elastomer with randomly distributed zwitterions). The zwitterion, urethane, and water molecules are represented by red, yellow, and blue shapes, respectively. Reproduced with permission [37], Copyright 2020, Elsevier B.V.

The self-healing mechanism driven by ionic interactions hinders the creation and disruption of intermolecular bonds within the network [37,59,60,61]. Self-healing materials based on ionic interactions have frequently been explored as a foundation for supramolecular hydrogels, mainly because of their high water solubility and robust electrostatic interactions. As an example of this self-healing mechanism, a network comprising a hydrocarbon backbone and acidic pendant groups, which are partially or fully neutralized to create salts that interact within the polymer network, was developed. These polar ionic groups generate electrostatic interactions, prompting the polymer chains to merge and mend cracks. Furthermore, incorporating small numbers of ionic groups leads to substantial enhancements in properties such as tensile strength, tear resistance, impact strength, and abrasion resistance. Several polymers derived from poly(ethylene-co-methacrylic acid) (EMAA) have been investigated. The EMAA is recognized for its sharpness, toughness, cutting ability, abrasion resistance, and chemical resilience. In addition, Varley et al. introduced self-healing ionic clusters based on EMAA neutralized with sodium ions [61]. EMAA forms an ionic cluster structure through interactions between the carboxylate anions and sodium cations, thereby creating regions of physical cross-linking. These ionic clusters provide networks with exceptional mechanical strength, even in the face of ballistic penetration. However, the restricted mobility of the network requires self-healing at elevated temperatures. Kang et al. mitigated this constraint by introducing water, which promotes the movement of ion clusters, thereby enabling self-healing at lower temperatures (Figure 10) [37].

#### 3.2.4. Hydrogen Bond Interactions

**Figure 11:**
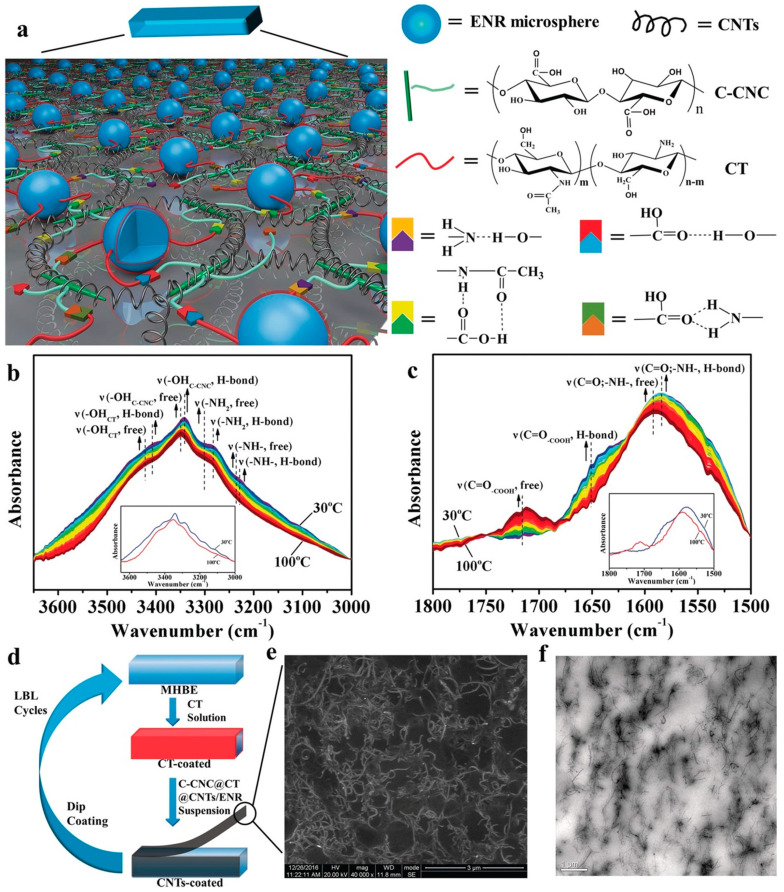
(**a**) Schematic illustration of proposed supramolecular networks formed by multiple hydrogen bonds, integrated with a nanostructured conductive network of carbon nanotubes (CNT) within epoxy natural rubber (ENR) latex microspheres. (**b**,**c**) Temperature-dependent FT-IR spectra of the carboxy cellulose nanocrystal and chitosan (C-CNC@CT) nanohybrid during heating from 30 °C to 100 °C: (**b**) 3650–3000 cm^−1^ and (**c**) 1800–1500 cm^−1^. The inset shows the FT-IR spectra of the C-CNC@CT nanohybrid at 30 °C (blue) and 100 °C (red). (**d**) Layer-by-layer (LBL) approach for strain sensor preparation. (**e**) SEM and (**f**) TEM images of the nanostructured C-CNC@CT@CNTs/ENR composites. Reproduced with permission [52], Copyright 2017, WILEY-VCH.

**Figure 12:**
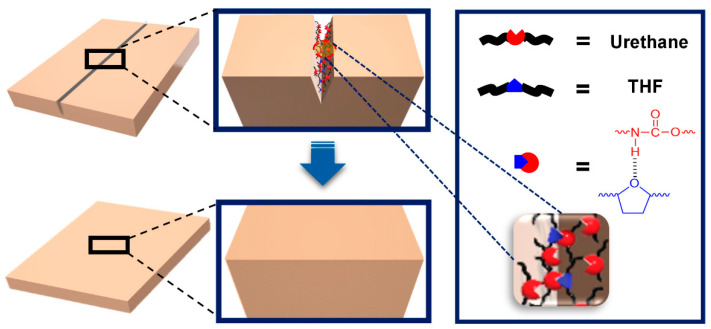
Graphical representation of the self-healing mechanism in polyurethane with heterocyclic moiety (PUT). The urethane group is depicted in red, while the heterocyclic group is shown in blue. Reproduced with permission [54], Copyright 2020, MDPI.

Hydrogen bonding is a straightforward and effective supramolecular interaction for achieving self-healing capabilities [12,52,53,54,55,56,57,58]. Hydrogen bonds are formed between a partially positive hydrogen atom bound to a relatively electronegative atom and the unpaired electrons of highly electronegative atoms (F, O, and N), with bond strengths ranging from 1 to 40 kcal/mol. The self-healing mechanism observed via temperature-dependent FT-IR spectroscopy is illustrated in Figure 11 [52]. As the temperature exceeds 100 °C, hydrogen bonds break, generating newly free groups. These disrupted hydrogen bonds are reestablished at the cut interfaces, resulting in rapid and highly efficient mechanical self-healing. The key focus of this research was to create multiple hydrogen-bonding networks by incorporating various groups capable of hydrogen bonding, thereby maximizing the strength of the hydrogen bonds. Figure 12 shows how the opportunity for hydrogen bonding was expanded using polyurethane as the polymer backbone [54]. Interestingly, introducing heterocyclic groups, which enhance hydrogen bonding, led to improvements in self-healing capabilities and mechanical strength.

Similar to polyurethane, polyurea was also explored by Li et al. [57]. They designed polyurea by dimerizing 2-ureido-4[1H]-pyrimidone (UPy) units. This study showed significant enhancements in Young’s modulus, tensile strength, and toughness when UPy units were integrated. UPy is a potent group capable of forming quadruple hydrogen bonds, which is a notable advancement considering that most hydrogen bonds consist of single to double bonds [58]. The hydrogen bonding-based self-healing system effectively reconciles the trade-off between self-healing and mechanical strength.

Consequently, the emergence of UPy groups with hydrogen bonds has prompted self-healing systems to incorporate hydrogen-bonded networks. Jin et al. show the addition of UPy and cyclodextrin structures into the polyurethane matrix [93]. The robust hydrogen bonding interactions of the UPy dimers served as the driving force for the self-healing performance, whereas the additional cyclodextrin formed a secondary cross-linked structure. This combination enhances the mechanical toughness and augments the overall self-healing performance.

In recent years, bio-based self-healing materials have emerged as promising in the development of new materials, combining sustainability with advanced functionality. Researchers have developed innovative materials capable of autonomous self-healing using renewable resources derived from biological origins. Biopolymers, including chitosan, cellulose, and proteins, distinguished by their capacity for hydrogen bonding, possess intrinsic biocompatibility and eco-friendly attributes. An example is the work of Zou et al., who employed biomass-derived gelatin and chitosan to engineer a self-healing electroactive bio-based hydrogel [94]. Incorporating amine and hydroxyl groups induces hydrogen bonding within the material, endowing it with exceptional self-healing properties. This biocompatible hydrogen bonding also imparts adhesive qualities suitable for various biological tissues.

Furthermore, integrating multi-walled carbon nanotubes imparts conductivity to the hydrogel, paving the way for its application in intelligent wearable sensors. Similarly, Shou et al. utilized bio-based polylactic acid, sourced from carbohydrates like corn, to synthesize a self-healing polyurethane [95]. This bio-based polyurethane demonstrates self-healing properties and exhibits excellent shape-memory effects. The material stands out for its high energy-dissipation values, a characteristic attributed to the formation of sacrificial hydrogen bonding interactions. Utilizing the self-healing potential of hydrogen bonds marks a significant advancement in enhancing the functionalities of bio-based materials.

#### 3.2.5. π–π Stacking Interactions

**Figure 13:**
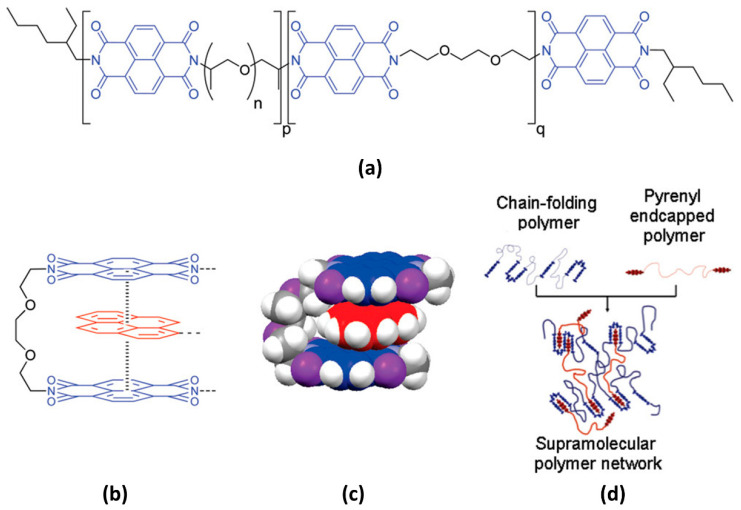
(**a**) Chemical structure of chain-folding polyimide. *n* is 6 on average, while p and q are approximately 7 each. (**b**) Proposed structure of the π–π stacking interaction between chain-folding polyimide and pyrenyl end-capped polymer. (**c**) Energy-minimized model of the electronically complementary π–π stacking interaction between bis(diimide) groups in chain-folding polyimide (denoted by segment q) and pyrenyl groups in bis(pyrenyl) end-capped polymer. (**d**) Schematic illustration of a supramolecular network established between chain-folding polyimide and a bis(pyrenyl) end-capped polymer. Reproduced with permission [97], Copyright 2010, American Chemical Society.

π–π stacking interactions occur when π electron-deficient groups interact with π electron-rich groups [96,97]. Most studies have detailed the formation of chain-folded assemblies involving typical electron-deficient diimide units and electron-rich pyrene derivatives. Burattini et al. presented a self-healing network comprising a polyimide-based chain-folding polymer and a pyrenyl end-capped polymer (Figure 13) [97]. When heat is applied, the chain-folded structure is disrupted, and electrostatic forces drive the interactions between the exposed cut surfaces. Additionally, Dai et al. employed pyrenyl groups and carbon nanotubes (CNTs) to develop a shape-memory polymer with self-healing capabilities [98]. With the assistance of π–π stacking interactions within the composites, self-healing properties were observed when exposed to near-infrared light. This paper clearly illustrates the rapid self-healing performance, with the recovery of the electrical properties within 150 s.

Li et al. demonstrated the fabrication of a self-healing conductive hydrogel, exploiting π–π stacking interactions, and explored its application as a strain and pressure sensor [99]. The polydopamine structure was constructed through the incorporation of dopamine, facilitating the formation of robust π–π stacking interactions. The introduction of acrylamide further enhanced the material by incorporating hydrogen bonds in addition to π–π stacking interactions. This dual-interaction mechanism was leveraged to propose strain and pressure sensors, capitalizing on resistance changes correlated with strain. The self-healing properties of the hydrogel, coupled with its intrinsic conductivity attributed to flexibility and carbon nanotubes (CNT), made it particularly effective for sensing applications. This showcases a multifunctional material with significant potential in flexible and self-healing electronics.

## 4. Applications of Self-Healing Materials

**Figure 14:**
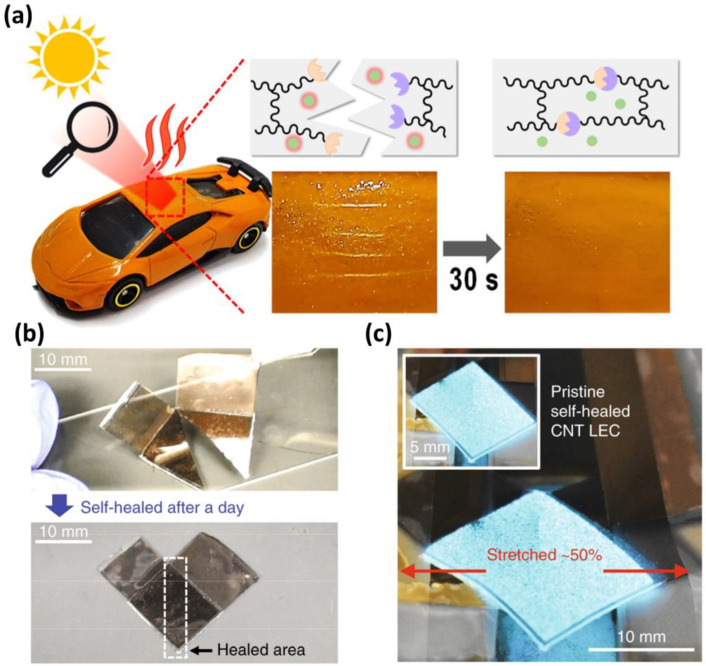
(**a**) Schematic illustration of the self-healing mechanism induced by near-infrared radiation. A self-healing polymer, employed as a protective coating on a model plastic automobile, exhibits rapid self-healing of a simulated scratch within 30 s upon exposure to focused sunlight. Reproduced with permission [101], Copyright 2022, American Chemical Society. (**b**) Photographs of a cut and self-healed (after a day) light-emitting capacitor (LEC) based on carbon nanotube (CNT) embedded self-healing polyurethane. (**c**) Stretch test after the self-healing process (after a day). Reproduced with permission [103], Copyright 2018, Springer Nature.

The practical application of self-healing materials is apparent in various consumer products and industries (Figure 14). An example is self-healing protective coating materials in electronic devices and automobiles [100]. These coating materials possess autonomous repair capabilities, effectively addressing surface damage. This augmentation significantly enhances the durability and lifespan of smartphones and tablets, preserving their initial appearances and ensuring sustained electrical performance over extended periods. In the high-end automotive industry, self-healing coating materials are exceptional in repairing surface damage resulting from road debris or parking incidents, thereby reducing maintenance expenses and preserving resale value [101]. Aerospace applications also gain advantages from self-healing materials: they maintain essential functions by repairing severe damage from bird strikes or abrasion during flight [102]. This not only enhances safety, but also reduces maintenance expenses for aerospace structures.

The initial applications of self-healing materials were focused on infrastructure and construction [104]. By incorporating these materials into concrete or sealants, structural elements can autonomously repair minor cracks and fissures, thus ensuring durability and sustained performance over time. These applications are crucial in preventing cracks from moisture infiltration, thus playing an essential role in ensuring building safety.

In the biomedical fields, self-healing materials have been used for advancing innovative medical devices [103,105]. These materials, including self-healing hydrogels and biocompatible substances, find a wide range of applications in drug delivery systems, tissue engineering, and implantable medical devices. Their ability to mend damage within the human body significantly contributes to the safety and effectiveness of biomedical applications.

Incorporating self-healing materials into energy applications, particularly lithium batteries and photovoltaics, represents a groundbreaking development [27,106]. In lithium-ion batteries, using self-healing materials in electrode coating can mitigate dendrite formation, extending the overall lifespan of batteries that power electronic devices and electric vehicles. Moreover, the self-healing properties effectively address microcracks and damage in electrode materials, thus maintaining battery efficiency and performance over numerous charge–discharge cycles. As energy applications increasingly pursue sustainable and resilient solutions, integrating self-healing materials holds immense promise for advancing the reliability and durability of energy storage technologies, paving the way for future innovations in these applications.

## 5. Conclusions

Self-healing properties have become increasingly indispensable in various industries, including automotive, electronics, optics, and architecture. Of particular significance is the potential to address contemporary environmental concerns, particularly the issue of plastic waste, by extending the lifespan of materials.

The evolution of self-healing materials has transitioned from extrinsic to intrinsic by harnessing the capabilities of dynamic covalent bonds and supramolecular interactions. Extrinsic-type self-healing offers immediate damage recovery but is constrained by limitations concerning the number of self-healing cycles it can undergo. Reversible chemical reactions and supramolecular interactions characterize intrinsic self-healing. Recent trends favor supramolecular interactions because they provide milder self-healing conditions. Nevertheless, challenges persist, particularly those related to mechanical strength and the demands of the self-healing process.

In pursuing an eco-friendly and sustainable future, self-healing must remain a focal point of research and development. Ongoing efforts are necessary to overcome these challenges and to ensure that self-healing materials continue to play a pivotal role in addressing environmental concerns and enhancing the durability of various industrial applications.

## Data Availability

Not applicable.
